# If painters give you lemons, squeeze the knowledge out of them. A study on the visual perception of the translucent and juicy appearance of citrus fruits in paintings

**DOI:** 10.1167/jov.20.13.12

**Published:** 2020-12-22

**Authors:** Francesca Di Cicco, Maarten W. A. Wijntjes, Sylvia C. Pont

**Affiliations:** 1 Perceptual Intelligence Lab, Faculty of Industrial Design Engineering, Delft University of Technology, Delft, The Netherlands; 2 Perceptual Intelligence Lab, Faculty of Industrial Design Engineering, Delft University of Technology, Delft, The Netherlands; 3 Perceptual Intelligence Lab, Faculty of Industrial Design Engineering, Delft University of Technology, Delft, The Netherlands

**Keywords:** translucency, juiciness, material perception, NMDS, image features, lemons, oranges, paintings

## Abstract

Citrus fruits are characterized by a juicy and translucent interior, important properties that drive material recognition and food acceptance. Yet, a thorough understanding of their visual perception is still missing. Using citrus fruits depicted in 17th-century paintings as stimuli, we ran three rating experiments. In Experiment 1, participants rated the perceived similarity in translucency or juiciness of the fruits. In Experiment 2, different groups of participants rated one image feature from a list obtained in a preliminary experiment. In Experiment 3, translucency and juiciness were rated. We constructed two-dimensional perceptual spaces for both material properties and fitted the ratings of the image features into the spaces to interpret them. “Highlights,” “peeled side,” “bumpiness,” and “color saturation” fit the juiciness space best and were high for the highly juicy stimuli. “Peeled side,” “intensity of light gradient,” “highlights,” and “color saturation” were the most salient features of the translucency space, being high for the highly translucent stimuli. The same image features were also indicated in a 17th-century painting manual for material depiction (Beurs, 1692; Beurs, in press). Altogether, we disclosed the expertise of painters with regard to material perception by identifying the image features that trigger a visual impression of juiciness and translucency in citrus fruits.

## Introduction

Eating is a multisensory experience. Often, the first interaction people have with food is visual, affecting their intentions to buy and consume a certain food. Several researchers have shown that vision can even affect taste perception by creating expectations (Piqueras-Fiszman & Spence, 2015). Food appearance drives quality perception and consumers’ acceptance, making it a fundamental problem for food industries and manufacturers. Despite its importance, though, this issue has seldom been approached from the perspective of vision to answer the question of how people visually estimate food material attributes. Here, we focused on understanding the perceptual spaces of the visual perception of translucency and juiciness for the case of citrus fruits.

Translucency and juiciness represent important quality parameters not only for citrus fruits but also for other types of fruits, such as apples (Harker, Amos, Echeverría, & Gunson, 2006), and even more so for meat (Winger & Hagyard, 1994). Juiciness is an especially important attribute that must be correct in meat and meat substitutes in order for them to be accepted (Elzerman, Hoek, van Boekel, & Luning, 2011).

Translucency and juiciness can be readily estimated from visual information. Although some research has been done on how we perceive translucent materials (Koenderink & van Doorn, 2001; Fleming & Bülthoff, 2005; Motoyoshi, 2010; Gkioulekas, Xiao, Zhao, Adelson, Zickler, & Bala, 2013; Marlow, Kim, & Anderson, 2017; Chadwick, Cox, Smithson, & Kentridge, 2018), no work to our knowledge has investigated the visual perception of juiciness. The features of local objects have been associated with translucency perception, especially edges and thin areas (Fleming & Bülthoff, 2005; Nagai, Ono, Tani, Koida, Kitazaki, & Nakauchi, 2013; Gkioulekas, Walter, Adelson, Bala, & Zickler, 2015) enhanced by back lighting (Xiao, Walter, Gkioulekas, Zickler, Adelson, & Bala, 2014). However, a thorough understanding of translucency perception of three-dimensional (3D) objects is still missing. Translucency is due to the complex optical phenomenon of subsurface scattering, and how it appears depends on the 3D shape of the object, the extinction coefficient of the medium (due to absorption and scattering), and the lighting and viewing directions (Koenderink & van Doorn, 2001). Although other optical properties such as color (Witzel & Gegenfurtner, 2018) and glossiness (Chadwick & Kentridge, 2015; Adams, Kucukoglu, Landy, & Mantiuk, 2018) have received more attention, and perceptual spaces have been constructed to relate bidirectional reflectance distribution function parameters to human perception of glossiness (Ferwerda, Pellacini, & Greenberg, 2001; Wills, Agarwal, Kriegman, & Belongie, 2009), the only attempt to find a perceptual embedding of translucency was done by Gkioulekas et al. (2013). In their study, they focused on the effect of the phase function (i.e., the angular distribution of light scattering) on the appearance of translucency to unravel which physical parameters can be used to elicit the desired translucent effect. Using classical multidimensional scaling (MDS), they found a two-dimensional (2D) space of translucency perception that could be well represented by the square of the average cosine of the phase function and by a function inversely related to the second moment of the cosine of the phase function. These corresponded, respectively, to a change in light diffusion and sharpness of the light gradient.

We assumed that the properties of translucency and juiciness are perceptually related. Juiciness is a complex food textural attribute dependent on the structure and the strength of the plant tissue (Mercado, Matas, & Posé, 2019) and corresponding to the amount and the rate of juice release during mastication (Szczesniak, 2002). Translucency is the optical phenomenon of light partially traveling through a medium, being scattered and then absorbed or transmitted. When light enters a citrus fruit, the juice contained in the vesicles making the pulp (McGee, 2004) is the scattering medium. A dry fruit would hardly appear translucent. Translucency and juiciness are also indicators of the fruit ripeness, and thus quality. Unripe oranges, for example, exhibit low transmittance (i.e., low translucency), and they contain the least amount of water (i.e., low juiciness) (Hussin, Wahid, Ahmad Hambali, Shahimin, Hasanuddin, & Azidin, 2017). Note that, in food science, the term “texture” refers to the mechanical and structural properties of food that are experienced on a multisensory level while eating (e.g., crispiness, stickiness, tenderness, juiciness) (Lu & Cen, 2013). This differs from the meaning of “texture” in vision science, where it is a statistically defined surface property of image regions (e.g., wavy, like water, like wood) (Landy & Graham, 2004). In art history, “texture” takes yet another meaning, referring rather indistinctly to all the material properties of a depicted object (e.g., shiny, rough). In this paper, we use the term “texture” as it is used in food science.

Our approach is to develop an understanding of the visual perception of material appearance by unraveling the image cues identified and exploited by painters to render different materials, based on the hypothesis that painters capture the triggers of material percepts, not necessarily realistically representing all optical details but phenomenologically depicting the key features. A painting is as ecologically valid as a photograph in representing reality, given that a photograph is a construction of lightings and viewpoints as much as a painting is. The same holds for computer renderings, the appearance of which is even more constructed, being totally controlled by the input parameters.

In this study, we sought to determine whether the visual perception of translucency and juiciness of citrus fruits rendered in 17th-century paintings could be embedded in perceptual spaces and in how many dimensions. We further aimed to identify which image features present in the paintings were used to estimate translucency and juiciness perception.

Several researchers have referred to realistic painters in order to understand the mechanisms of human visual perception (Adelson, 2001; Koenderink & van Doorn, 2001; Wijntjes, Doerschner, Kucukoglu, & Pont, 2012; Marlow et al., 2017; Barla, 2017; Casati & Cavanagh, 2019). Painters have been regarded as “early vision scientists” (Sayim & Cavanagh, 2011), because the way they represent the world taps into the processes of the human visual system via abbreviations of the laws of physics (Cavanagh, 2005). The field of computer graphics is also turning toward more art-based and perception-driven approaches (Khan, Reinhard, Fleming, & Bülthoff, 2006; Schmidt, Pellacini, Nowrouzezahrai, Jarosz, & Dachsbacher, 2014; Bousseau, 2015), given the human vision tolerance for some physical inaccuracies (Bertamini, Latto, & Spooner, 2003; Mamassian, 2004; Ostrovsky, Cavanagh, & Sinha, 2005), and to avoid the computational costs (Ferwerda, 2003) and artificial, too perfect look of physically based renderings (Yan, 2018). Moreover, using simplified depictions containing just perceptual triggers and ignoring what the visual system is insensitive to might also enhance experience. For example, as suggested by Parraman (2014), the highly convincing representation of material attributes achieved by painters from the 15th century on can be ascribed to their economical and almost gestural brushworks, as “too much information possibly hinders the appearance.”

Dutch painters from the 17th century were masters at the expression of stuff (De Vries, 1991). Cut-open lemons and oranges that revealed their juicy and translucent insides became a recurring motif in Dutch still-lifes, particularly after Pieter Claesz (1597–1661) painted a peeled lemon in the second decade of the 17th century (Westermann, 2017). Lemons and oranges, like many other objects in the 17th century, were painted according to standard, systematic recipes (Wallert, 1999; Wallert, 2012; Wiersma, 2019). Instructions of this kind can be found in a 17th-century manual, *The Big World Painted Small*, written by Willem Beurs (Beurs, 1692; Beurs, in press). This manual provides a collection of shortcuts to render the optical behavior of materials by tweaking features of their highlights, such as color, contrast, or sharpness. Parametric changes of such image features have been shown to affect not only gloss perception (Marlow & Anderson, 2013) but also the perceived material category (Schmid, Barla, & Doerschner, 2020). In previous work, Beurs’ manual has supported the notion that contrast and blurriness, but not coverage of highlights, were the image features used to render the glossiness of grapes in 17th-century paintings. The grapes recipe contained in the manual also confirmed the artistic convention of using white to render highlights, thus providing an example of using key perceptual information to produce an efficient yet effective rendering of material properties (Di Cicco, Wijntjes, & Pont, 2019). We also considered Beurs’ recipes for additional insights into the image features and perceptual shortcuts exploited by painters to render translucency and juiciness.

## Methods

### Overview

The study consisted of three parts. In the first part (Experiment 1), we ran two similarity rating experiments, one on juiciness and one on translucency. In the second part (Experiment 2), participants each rated one of seven features from a list collected during a questionnaire in a preliminary experiment. The features were rated to develop a meaningful interpretation of the perceptual spaces of translucency and juiciness. In the last part (Experiment 3), participants rated the translucency and juiciness of all of the stimuli.

### Stimuli

**Figure 1.**
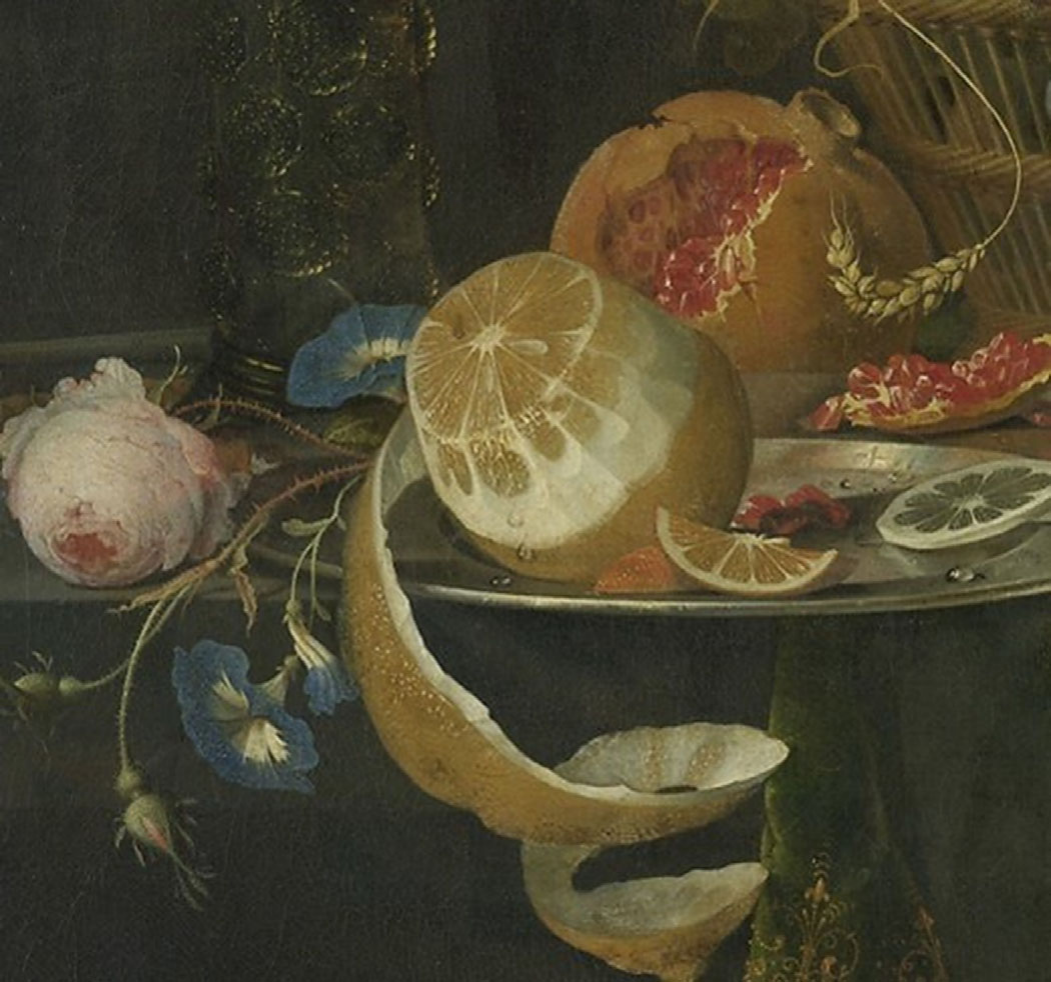
Example of a stimulus presentation as a squared cutout containing the target citrus. Abraham Mignon's *Still Life with Fruit and a Goldfinch* (1660–1679) was downloaded from the online repository of the Rijksmuseum, Amsterdam.

The stimuli consisted of 55 digital images of 17th-century paintings depicting citrus fruits. The images were downloaded from the online repositories of several museums and collections. The stimuli were presented on the screen as cutouts containing the target citrus and part of the background, as shown in Figure 1. (See also Supplementary Figure S1 for a list of all of the squared cutouts used in the experiments; each image in the list is linked to the relative museum repository website where the original image can be found.) To ensure that the visual size of the citrus fruits was kept consistent between stimuli, the cutouts were made so as to keep a constant ratio between the width of the pulp and the width of the resulting image.

### Observers

Two groups of seven and six observers participated in Experiment 1, one group rated the similarity in translucency and the other group the similarity in juiciness. The participants were students recruited via email within Delft University of Technology. Experiments 2 and 3 were conducted on Amazon Mechanical Turk (AMT). Seven different groups of 10 participants each took part in Experiment 2, and two different groups of 10 participants each participated in Experiment 3.

All participants were naïve to the purpose of the experiments. They agreed with the informed consent prior to the experiment and received compensation for their participation. The experiments adhered to the tenets of the Declaration of Helsinki and were approved by the Human Research Ethics Committee of the Delft University of Technology.

### Procedure experiment 1

Experiment 1 was conducted online using p5.js (McCarthy, Reas, & Fry, 2015). A link to the code of the experiment was sent to the participants via email. They also received video instructions in which they were shown all the images before starting the experiment to gain an overview of the stimuli range. Next in the instructions they were provided with a written definition of the attribute to rate (see Supplementary Material S1) and an explanation of the task and the polarity of the scale (1 = low similarity; 7 = high similarity). At the end of the experiment, participants could automatically download the data, which they sent back to the experimenter via email.

The task was to rate on a continuous seven-point scale the similarity of either the translucency or the juiciness of two fruit pulps. The 55 stimuli provided a total of 1485 pairs of images, which were rated once. The trials were randomized across participants. The question shown on the screen was, “How similar is the [attribute] of the pulps of these citrus fruits?” In the instructions, participants were explicitly told to focus on the pulps only and to avoid basing their judgments on similarities in shape or orientation of the whole fruit.

### Procedure experiment 2

Experiment 2 consisted of rating a list of image features obtained from a questionnaire conducted during a preliminary experiment in the lab. In the preliminary experiment, two groups of six participants each rated the similarity in juiciness or translucency for a subset of 38 stimuli. After they finished doing that, they were asked to fill in a questionnaire with two questions: “Describe how you rated the similarity of the [attribute] of the pulps” and “Which features of the object did you use?” During completion of the questionnaire, prints of all of the stimuli were available for the participants so they could point out areas with specific stimuli to the experimenter. The answers to the questionnaire were evaluated by the authors via frequency analysis (data not shown), and they were used to generate a list of image features that might relate to the perceptual spaces of translucency and juiciness. The list included intensity of the light gradient, sharpness of the light gradient, color saturation, surface bumpiness, highlights, visible seeds, and peeled side of the pulp. In this study, we sought to distinguish among the physics, the (pictorial) representation, and the visual perception of material properties. They are closely related but not exclusively determined by physics. Optical properties such as translucency can most directly be visually interpreted, and thus perceived, based on the features of the image structure rather than by retrieving exact physical parameters. We tested the perception of such image features in Experiment 2.

The experiment was conducted online on AMT. The selection criteria for participants was an approval rate of minimum 95% over at least 1000 completed tasks. Participants were randomly assigned to one of the seven features. The order of the stimuli was randomized across participants. Prior to the experiment, participants were shown all of the images to gain an overview of the stimuli range. Afterward, they received written instructions regarding the question, the definition of the feature to rate, and the explanation of the scale polarity (see Supplementary Material S2). “Intensity of the light gradient,” “sharpness of the light gradient,” “color saturation,” and “bumpiness” were rated on a continuous seven-point scale, three times for each of the 55 stimuli for a total of 165 trials per task. The features “highlights,” “peeled side,” and “visible seed” were judged via yes/no questions. The three yes/no questions were answered once for each of the 55 stimuli.

Hereafter, for readability, we refer to the ratings of the image features simply by the term for the feature (e.g., referring to the rating of the intensity of the light gradient as the “intensity of gradient”), but please note that these all concern perceptual ratings and not actual image measures.

### Procedure experiment 3

The procedure for Experiment 3 was the same as for Experiment 2 (for the instructions, see Supplementary Material S3). Participants on AMT rated either translucency or juiciness on a continuous seven-point scale, three times for each of the 55 stimuli for a total of 165 trials per task.

## Results

### Internal consistency

To analyze the internal consistency among participants for all of the experiments, we normalized the data of each participant, rescaling to a range of 0 to 1 to account for possible effects of unequal interval judgments. The yes/no data on the presence of highlights, seeds, and a peeled side were converted to yes = 1 and no = 0.

For Experiment 1, the inter-rater agreement, calculated as the mean correlation of the ratings of all observers, was *r* = 0.51 (*p* < 0.05) for translucency and *r* = 0.53 (*p* < 0.05) for juiciness. In Experiment 2, the features “intensity of gradient,” “sharpness of gradient,” “color saturation,” and “bumpiness” were rated three times per stimulus. To smooth out the effects of potential outliers we took the median over the three repetitions. The mean intra-rater correlations ranged from 0.65 to 0.89 (*p* < 0.001) for intensity of gradient, from 0.64 to 0.85 (*p* < 0.001) for sharpness of gradient, from 0.39 to 0.66 (*p* < 0.05) for color saturation, and from 0.47 to 0.71 (*p* < 0.01) for bumpiness. The agreement among participants was *r* = 0.7 (*p* < 0.001) for intensity of gradient, *r* = 0.73 (*p* < 0.001) for sharpness of gradient, *r* = 0.44 for color saturation (*p* < 0.05), and *r* = 0.62 (*p* < 0.05) for bumpiness.

The three yes/no questions about the presence of highlights on the pulp surfaces and of seeds in the pulps and whether the citrus fruits were peeled, showing the pulp on the side, were answered once per stimulus. Fleiss’ kappa showed that there was moderate inter-rater agreement on the presence of visible seeds (κ = 0.47, *p* < 0.001) and of highlights (κ = 0.43, *p* < 0.001), and there was substantial agreement on the presence of the peeled side (κ = 0.75, *p* < 0.001).

Finally, the intra-rater agreement in Experiment 3 ranged from 0.57 to 0.77 (*p* < 0.001) for translucency and from 0.66 to 0.77 (*p* < 0.001) for juiciness. The inter-rater agreement was *r* = 0.66 (*p* < 0.001) for translucency and *r* = 0.67 (*p* < 0.001) for juiciness.

Overall, the agreement among participants in the three experiments was at a level that allowed for further analysis.

### Dimensionality of the perceptual spaces of translucency and juiciness

The similarity data for Experiment 1 were analyzed via non-metric multidimensional scaling (NMDS). NMDS represents similarity data, in general proximities, in a new configuration with the least possible number of dimensions to achieve the best fit while still reproducing the distances of the data as closely as possible. NMDS addresses the limitations of applying metric MDS to human rating data, in that it does not rely on the magnitude of the dissimilarities but rather on their rank order (Shepard, 1962). Thus, the reason for using NMDS was to handle perceptual data whose actual distances are unknown.

The analysis was run using the function metaMDS from the vegan package (v2.5-5) in R (R Foundation for Statistical Computing, Vienna, Austria; Oksanen, Ueda, & Spence, 2019). The similarity ratings were converted to dissimilarity distance matrices by subtracting the ratings from 1.

**Figure 2.**
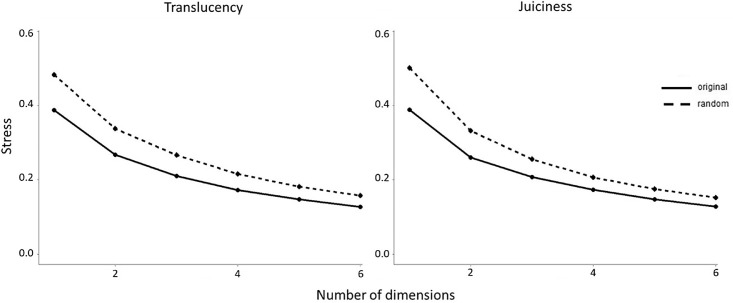
Scree plots showing the stress values as a function of the number of dimensions. The solid line represents the scree plot for the original values, and the dashed line shows the scree plot of the random data obtained from the average of the permutations. (Left) Scree plot for translucency NMDS space. (Right) Scree plot for juiciness NMDS space.

To determine the dimensionality of the translucency and juiciness spaces, we calculated the stress as defined by Kruskal (1964) for one-dimensional to six-dimensional configurations. The resulting scree plots are shown in Figure 2.

One criterion for choosing the optimal number of dimensions is to look for an “elbow” in the scree plot (i.e., a steep decrease of stress followed by a plateau), which indicates that the addition of dimensions to the space would just fit noise and not significantly reduce the stress. Our scree plots do not show a clear elbow, as is often the case with human data (Borg, Groenen, & Mair, 2018). Another approach is to pick the number of dimensions that allow for a stress value below 0.2, indicating an adequate fit (Kruskal, 1964). The stress values for two dimensions were 0.27 for translucency and 0.26 for juiciness, thus higher than the threshold of 0.2 proposed by Kruskal (1964). However, the appropriateness of the strict cutoff at 0.2 has been questioned by several researchers. Borg et al. (2018) stated that, “An MDS solution can be robust and replicable, even if its stress value is high. Stress, moreover, is substantively blind; i.e., it says nothing about the compatibility of a content theory with the MDS configuration, or about its interpretability.” The stress value depends on several factors, including the number of points, the number of dimensions, and the amount of noise in the data (Borg & Groenen, 2005). Dexter, Rollwagen-Bollens, and Bollens (2018) proposed a permutational-based null model for the evaluation of the stress. According to this model, we generated 100 permutations for the similarity matrices of translucency and juiciness; we then calculated the stress values for these random datasets and compared them with the stress of the original data. The scree plots for the original data (solid line) and the random data (dashed line) are compared in Figure 2. A *t*-test showed that the stress values obtained for the original data were significantly (*p* < 0.001) different from the random ordinations. We can thus conclude that the 2D configurations contain some meaningful structure. We further analyzed the dimensionality according to the criterion of interpretability of the coordinates proposed by Kruskal (1964). We compared, via visual inspection, the distribution of the stimuli in 2D and 3D spaces for both translucency and juiciness. Because the third dimension did not reveal any further structure, we opted for the 2D space in both cases.

### Interpretation of the perceptual spaces of translucency and juiciness

**Figure 3.**
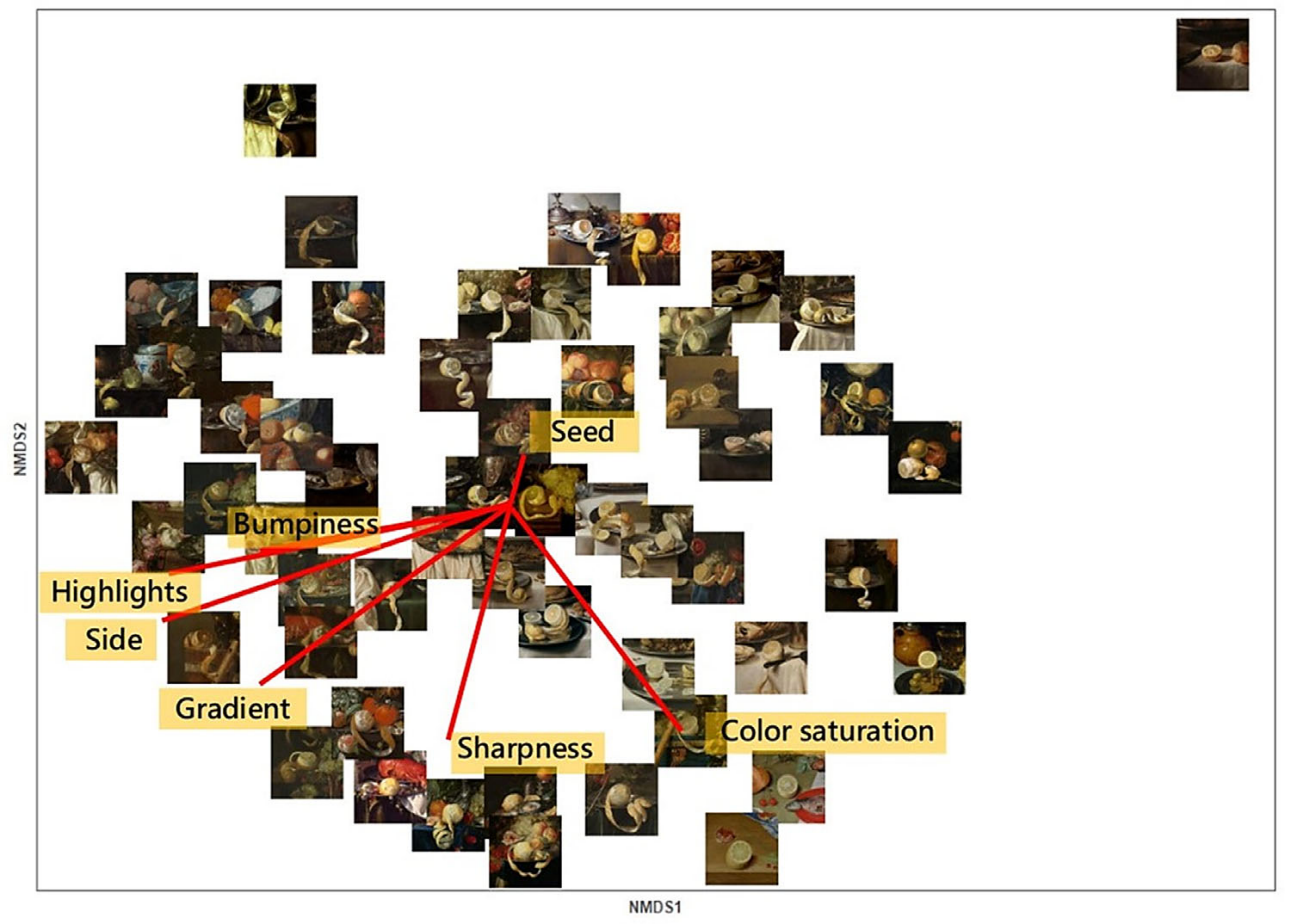
2D space of translucency perception with the stimuli shown at the corresponding coordinates. The red lines represent the vectors of the image features fitted in the space.

**Figure 4.**
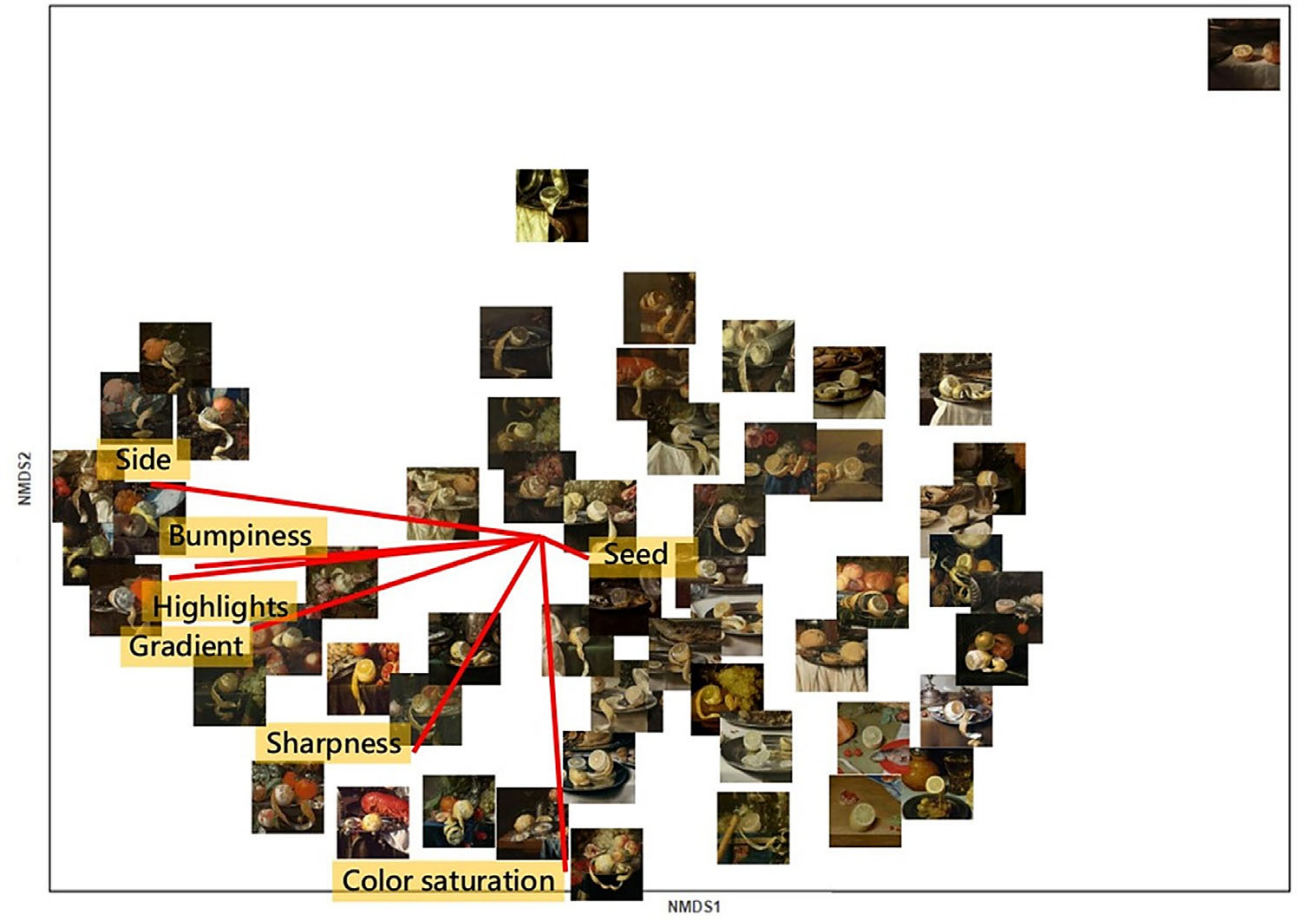
2D space of juiciness perception with the stimuli shown at the corresponding coordinates. The red lines represent the vectors of the image features fitted in the space. The space was rotated using Procrustes analysis for better comparison with the ordination of the stimuli in the translucency space in Figure 3.

Figures 3 and 4 show the 2D embeddings of the perceived similarities of translucency and juiciness, respectively, together with the vectors of the features fitted onto the spaces. The ordination of the juiciness space shown in Figure 4 was matched via Procrustes analysis to the translucency space shown in Figure 3. The significance of the Procrustes result was tested by permutation, resulting in high and significant correlation between the two ordinations (*r* = 0.78, *p* < 0.001).

To interpret the underlying structure of the multidimensional spaces, we performed property vector fitting (Perkins & Reynolds, 1995). For property vector fitting, we used the function envfit from the vegan package (v2.5-5) in R (Oksanen et al., 2019) to fit vectors of the features rated in Experiment 2 onto the spaces, such as to maximize the correlations of the projections of the scores onto the vectors with the corresponding rated features. The length of the vectors illustrates the strength of the correlation, and the orientation indicates the direction that maximizes the correlation.

We computed the correlations given by the vector fitting to interpret the configurations of the translucency and juiciness spaces. The projections of the scores were calculated as the distance *d_i_* from the origin to the scores projected onto the vectors, using the following formula (Bergmann Tiest & Kappers, 2006):

(1) $$d_{i}=\frac{\vec{p}\cdot {\vec{x}}_{i}}{\left|\vec{p}\right|}$$

where $\vec{p}$ is the vector of a rated feature, and ${\vec{x}}_{i}$ is the score representing a stimulus in the NMDS space. The correlation coefficients between the features rated in Experiment 2 and the projections of the scores onto their vectors, together with their significance level, are reported in Table 1. All image features showed high and significant correlation with both spaces, except for the presence of seeds in the pulp, which did not correlate with either of the spaces. In Table 2, we report the correlations between the ratings of translucency and juiciness from Experiment 3 with the ratings of the image features. The stimuli rated most translucent and juicy in Experiment 3 also had high values of intensity and sharpness of the light gradient; the images showed fruit that was peeled on the side, was bumpy, and had highlights, as shown by the positive and significant correlations in Table 2.

**Table 1.** Correlations between the distance of the scores projected onto the vector of each attribute and the corresponding ratings from Experiment 2 in the 2D translucency space (first column) and in the 2D juiciness space (second column). *Note:* *p < 0.05; **p < 0.01; ***p < 0.001.

	Scores	
	Translucency	Juiciness
Intensity gradient	0.64***	0.59***
Sharpness gradient	0.49***	0.50***
Color saturation	0.58***	0.69***
Bumpiness	0.50***	0.59***
Highlights	0.70***	0.72***
Peeled side	0.77***	0.77***
Visible seeds	0.08	0.12

**Table 2.** Correlations between the ratings of the features from Experiment 2 with the ratings of translucency (first column) and juiciness (second column) from Experiment 3. *Note:* *p < 0.05; **p < 0.01; ***p < 0.001.

	Translucency	Juiciness
Intensity gradient	0.65***	0.41**
Sharpness gradient	0.31*	0.42**
Color saturation	0.10	0.17
Bumpiness	0.40**	0.38**
Highlights	0.61***	0.61***
Peeled side	0.70***	0.65**
Visible seeds	–0.11	0.17

## Discussion

In this study, we first aimed to determine the dimensionalities of the perceptual spaces of human visual perception of translucency and juiciness of citrus fruits pulps depicted in 17th-century paintings. Second, we intended to identify and evaluate the perceptual relevance of image features found in the paintings for interpretation of the spaces.

We found that 2D embeddings were the optimal solutions for both perceptual spaces, based on the evaluation of the stress values compared to random configurations. We further relied on the criterion of interpretability of the coordinates proposed by Kruskal (1964) to opt for the 2D solutions, given that a visual inspection of the third dimension of both spaces did not lead to additional understanding of translucency and juiciness perception.

We assumed that the translucency and juiciness spaces were perceptually related, and we found that similar features were associated with the perception of both attributes. Procrustes analysis showed that the ordination of the stimuli was similarly above chance between the translucency and the juiciness spaces, demonstrating the robustness of the underlying structures of the data.

The interpretation of the two dimensions of the spaces was drawn from vector fitting of the image features rated in Experiment 2 and by correlating the ratings of translucency and juiciness in Experiment 3 with the ratings of the features.

The norm of the vectors represented the importance of each image feature for the perceptual judgment of translucency and juiciness. The vectors that best fitted the translucency space were the presence of highlights on the pulp, the peeled side, the intensity and sharpness of gradient, and color saturation.

Because we tested a limited and specific set of stimuli, we cannot draw conclusions about the space of translucency perception that can be generalized to every translucent material. Different translucent materials might require additional dimensions and features to fit into the space. Nonetheless, the list of image features that we used to interpret our translucency space of citrus fruits agreed with previous research on translucency perception. Fleming and Bülthoff (2005) compiled a list of image features that they found contributed to the visual appearance of translucency; they used computer-rendered stimuli to evaluate a bidirectional scattering surface reflectance distribution function (Jensen, Marschner, Levoy, & Hanrahan, 2001). Their list includes highlights, color saturation, important image regions, image contrast, and blur. Image contrast and image blur correspond to our intensity of gradient and sharpness of gradient, and the important image regions can be related to what we referred to as the “peeled side,” which is an image feature specific for citrus fruits. The pulp also being visible from the side allows easy perception of the light bleeding through the edges of the object (Gkioulekas et al., 2015), thus increasing the translucent impression. The relationship between the light gradient and translucency perception was also found by Wijntjes, Spoiala, and de Ridder (2020) for the case of sea waves depicted in paintings.

Gkioulekas et al. (2013) proposed a two-dimensional perceptual space for translucency corresponding to two parameters of the phase function that mainly affect light diffusion and sharpness. These may be qualitatively related to what we refer to as “intensity of gradient” and “sharpness of gradient,” which we also found to be important parameters for the ordering of stimuli in the translucency space, but not independent dimensions. However, it is difficult to draw a direct comparison with their study, given the essential difference in their choice of well-controlled computer rendered objects as stimuli. By using totally uncontrolled stimuli such as paintings, we allowed for variations across a wide range of (unknown) features. This may have disclosed different relationships among perceptual dimensions.

**Figure 5.**
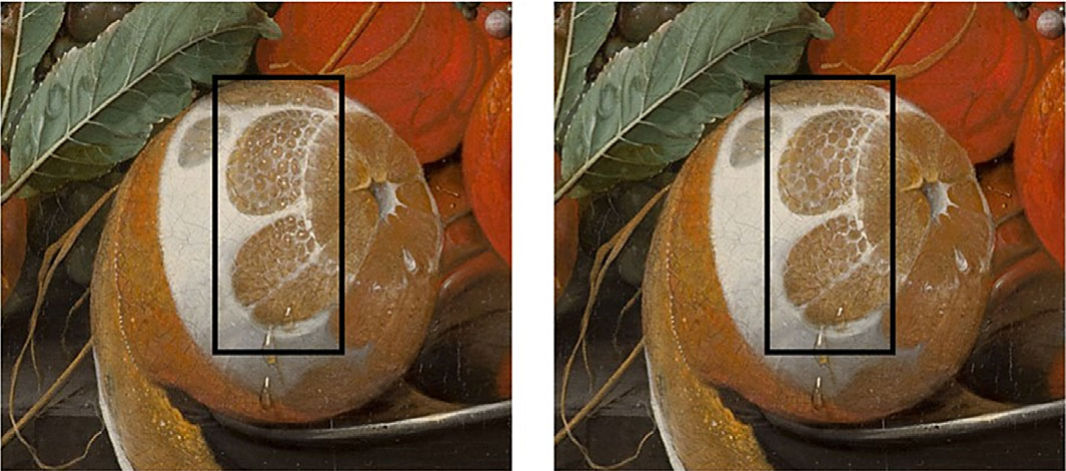
Detail of the stimulus perceived to be the bumpiest. The black box indicates the part that was manually modified by the authors to remove the highlights. (Left) Original painting; (right) modified version without highlights on the side of the pulp. Cornelis de Heem's *Fruit Still Life* (1670) was downloaded from the online repository of the Mauritshuis, The Hague.

The present study is the first, to our knowledge, to investigate the visual perceptual space of juiciness. The vectors that best fitted the juiciness space were the peeled side, presence of highlights, bumpiness, intensity of gradient, and color saturation. Bumpiness, together with the presence of highlights and a peeled side, were oriented toward the first dimension of the juiciness space (Figure 4). The metaMDS function that was used to construct the space also rotated the configuration to maximize the variance of the points along the first dimension (Oksanen et al., 2019), meaning that these features were the most salient to sort juiciness perception. The bumpiness of the pulp surface is a straightforward indication that the cells are full of juice. A peeled side allows better perception of whether the cells of the pulp are swollen and bumpy or empty and flat. The presence of highlights provides additional information to perceive the 3D shape of the pulp (Norman, Todd, & Orban, 2004; Ho, Landy, & Maloney, 2008), hence the bumpiness. These three image features can all be observed in the pulp of the fruit that was perceived to be the juiciest; it was also the bumpiest, had a peeled side, and was among the fruit with the most highlights (Figure 5, left). All of the small white dots mimicking highlights on the peeled side give the fruit a stronger 3D appearance compared to the same image from which the highlights have been removed (Figure 5, right).

**Figure 6.**
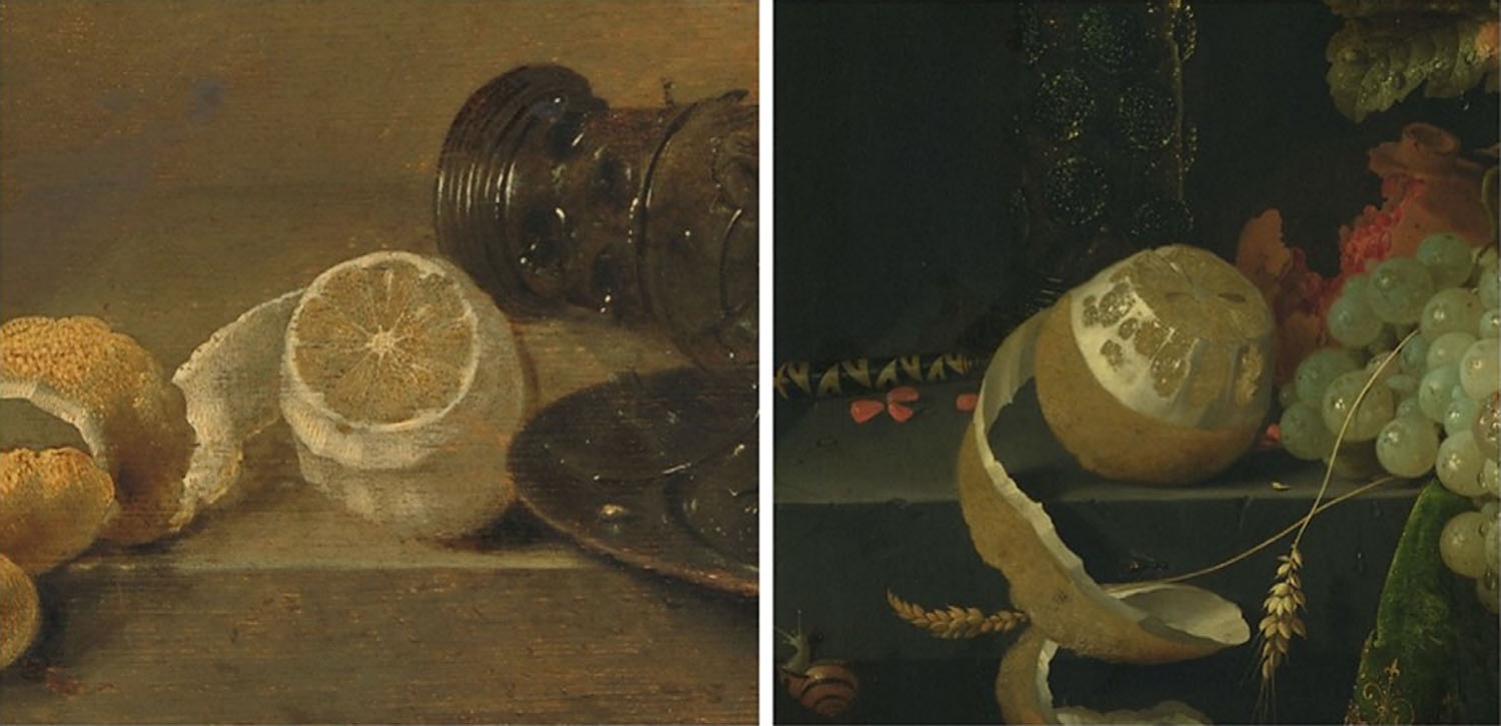
Examples of two stimuli with the seeds visible inside the pulp. The one on the left was perceived to be among the least translucent and least juicy, whereas the one on the right was rated as being highly translucent and juicy. (Left) Willem Claesz Heda, *Still Life with a Broken Glass* (1642); (right) Abraham Mignon, *Still Life with* *Fruit* *and a Beaker on a Cock's Foot* (1660–1679). The images were downloaded from the online repository of the Rijksmuseum, Amsterdam.

Among our list of image features, only the visible seeds seemed to not contribute to the interpretation of the perceptual spaces of translucency and juiciness. A visual inspection of the stimuli showed that seeds could be visible in pulps with a dry and non-translucent appearance (Figure 6, left), as well as in translucent and juicy pulps (Figure 6, right). Even though visible seeds were not found to be a cue, it was probably reported by our participants because the property of seeing inner parts is often associated with transparent and translucent media.

The value of the implicit knowledge of painters regarding material perception has been widely recognized (Adelson, 2001; Koenderink & van Doorn, 2001; Sayim & Cavanagh, 2011; Wijntjes et al., 2012; Marlow et al., 2017; Barla, 2017), but the actual use of paintings as stimuli to research and understand how we perceive material properties is novel and still in its infancy (Di Cicco et al., 2019; Van Zuijlen, Pont, & Wijntjes, 2020; Wijntjes, et al., 2020). Our approach was also new in that we measured the perceived similarity of a specific material property (either translucency or juiciness) to reveal the complexity of its perception. For example, by correlating the ratings of translucency and juiciness from Experiment 3 with the ratings of the image features from Experiment 2 (Table 2), we observed that neither translucency nor juiciness was correlated with color saturation (*r* = 0.1 for translucency and *r* = 0.17 for juiciness; both *p* > 0.05). However, the vector fitting in their 2D perceptual spaces (Figures 3 and 4) revealed that color saturation could be identified with the second dimension of both spaces. As argued by Fleming and Bülthoff (2005), even though color saturation can have an effect it is neither necessary nor sufficient to trigger a translucent impression. Nonetheless, color saturation was spontaneously reported in the questionnaire by participants of the preliminary experiment, and we found that the stimuli were consistently ordered along such higher dimensions.

The psychophysical measurements of the features used to interpret the spaces might be considered a limitation of this work. We believe, however, that this approach is justified by the nature of some of our features (bumpiness, presence of highlights, peeled side, visible seeds), which were distal visual cues that cannot be easily and correctly quantified via image analysis. Image statistics, such as skewness (Motoyoshi, Nishida, Sharan, & Adelson, 2007), have been shown to not be adequate predictors of surface reflectance properties, as they fail to take into account the consistency between the perceived 3D shape and the positions and orientations of highlights on the surface (Anderson & Kim, 2009). As argued by Wijntjes et al. (2020), quantifying the visual cues from the image without considering the 3D shape and the lighting environment would be meaningless. The other three features on our list—magnitude and sharpness of the light gradient and color saturation—could be measured via image analysis, but, again, the measurement would not be complete. Especially in the case of the light gradient, other factors beside the change in the luminance values play a role, such as the shading pattern and its distribution around the pulp. This effect would have to be calculated by disentangling the material, shape, and lighting effects, but to our knowledge no algorithm can do that yet.

Finally, the measurement of the features via image analysis would have to be proven valid via correlation with the psychophysical estimations.

### Beurs’ instructions on the material properties of lemons and oranges

Historical painting instructions are a great source of information, not only for the purpose of studying technical art history (Lehmann, 2007; Stols-Witlox, 2017) but also to complement the implicit perceptual knowledge inherent in paintings. For example, Lehmann, Pont, and Geusebroek (2005) investigated the texture appearance of tree bark and foliage, combining the findings that Leonardo da Vinci reported in his *Trattato della Pittura* with computer vision and ecological optics to understand tree depictions.

**Figure 7.**
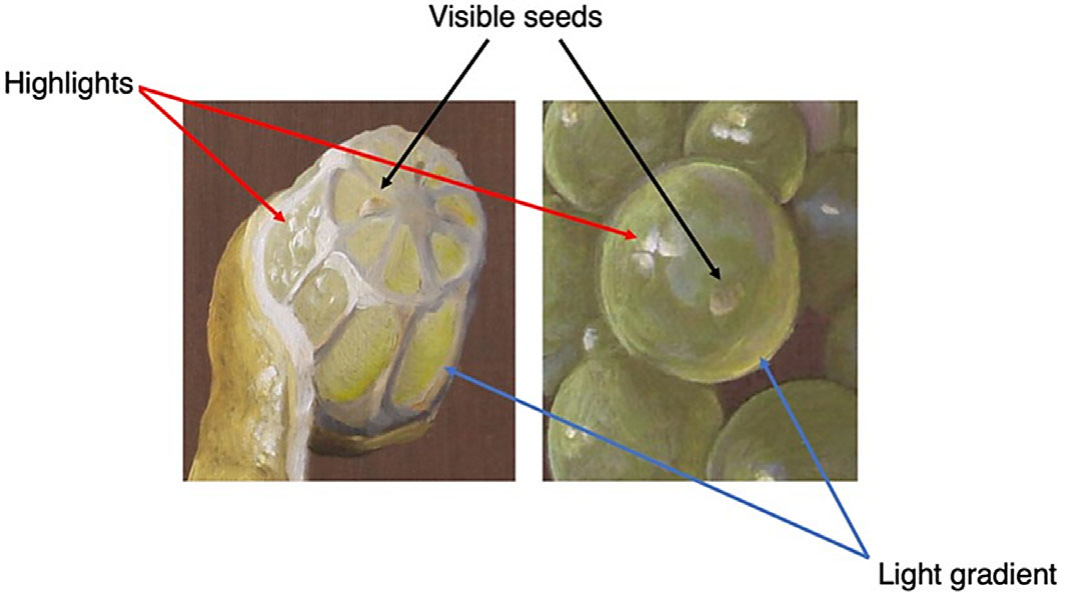
Visualization of how the pictorial recipe of the grapes was reapplied to render the pulp of a lemon according to Beurs’ recipe. The image features explicitly addressed by Beurs are marked with an arrow. (Paintings by Lisa Wiersma.)

Here, we referred to the painting manual *The Big World Painted Small* (Beurs, 1692; Beurs, in press), which is a collection of pictorial recipes for rendering objects and materials in the most convincing way using oil paint. The book has a descending structure, in which the basics are explained and practiced in the beginning and there is no need to repeat them in every recipe; the same is valid for similarities between materials. That is why Beurs’ instructions on how to paint the pulp of a lemon or an orange consist of a series of references to previous recipes, including grapes, the first food treated in the book (Beurs, 1692; Beurs, in press). Beurs aimed to teach how to paint materials rather than objects, so he observed that the techniques necessary to recreate the composition of the layers of grapes could be reapplied to painting gooseberries, oranges, and lemons. From the grapes recipe, we could derive the image features prescribed to render citrus fruit pulps—the light gradient, placing the highlights opposite the brighter contours along the edges, and the visible seeds (Figure 7). Beurs also implicitly referred to the use of bright colors when listing the color pigments to employ.

**Figure 8.**
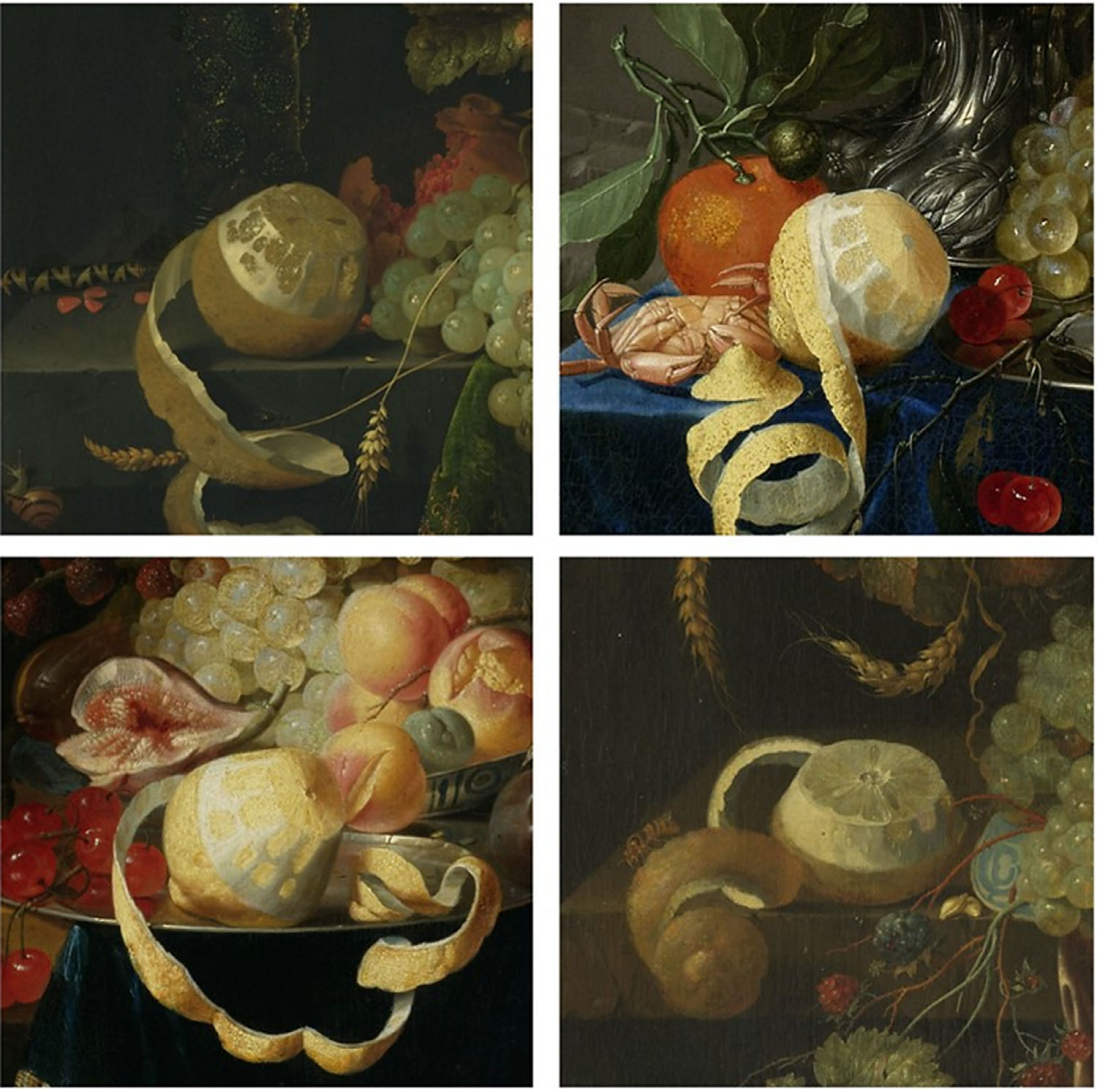
Examples of stimuli peeled on the side and showing top-left lighting and bottom-right shading. The shade on the white pith increases the contrast with the pulp, making it appear lighter. (Top left) Abraham Mignon, *Still Life with Fruit and a Beaker on a Cock's Foot* (1660–1679); (top right) Pieter de Ring, *Still Life with Golden Goblet* (1640–1660); (bottom left) Johannes Hannot, *Still Life with Fruit* (1668); (bottom right) Jan Davidsz. de Heem, *Still Life with Fruit and a Lobster* (1640–1700). All of the images were downloaded from the online repository of the Rijksmuseum, Amsterdam.

Given that in still-life paintings the light source is conventionally placed top left (Mamassian, 2008), the lighter part of the gradient is usually painted at the bottom right of the pulp. Such top-left lighting also means that the bottom right side of the lemon is shaded, and when the side is peeled the contrast between the pulp and the white pith of the citrus fruit increases. Such contrast produces an appearance even lighter along the edges of the fruit pulp, confirming the importance of a visible light gradient through the pulp to trigger an impression of translucency (for examples from our stimulus set, see Figure 8).

## Conclusions

In this study, we determined that the optimal embeddings for the perception of translucency and juiciness of the pulps of citrus fruits depicted in 17th-century paintings are two dimensional. We then identified the image features that provide a perceptually meaningful interpretation of these spaces. We assumed a perceptual relationship between translucency and juiciness, and we found that similar image features were related to their perceptual spaces. The present study is the first, to our knowledge, to investigate the visual space of juiciness perception, a food textural attribute usually studied in relation to in-mouth perception (Boulton, Corrigan, & Lill, 1997) and physical measurements for fruit quality determination (Guthrie, Walsh, Reid, & Liebenberg, 2005; Riaz et al., 2015). Visual perception is known to affect the overall sensory experience of food, but the effect of the visual perception of food textural properties is still unknown (with the exception of Okajima et al., 2013). Thus, identifying the visual dimensions that people use to infer the textural properties of food can advance our current understanding of human multisensory perception of food. Our findings could contribute to the fields of human–food interaction influenced by the visual appearance of food, such as expectations of liking and flavor (Hurling & Shepherd, 2003), eating behavior (Wadhera & Capaldi-Philips, 2014), and purchase intentions (Imram, 1999).

Finding image features that are perceptually significant to trigger perceptions of specific material properties could also be beneficial for computer graphics. Working with scientifically informed, perception-based visual cues, such as the ones found in this study, could reduce the time spent on trial and error, allowing researchers to adjust the parameters as necessary to obtain the desired appearance.

The translucency space was interpreted via image features that agreed with previous literature (Fleming & Bülthoff, 2005) and may thus be generalized to light gradient, highlights, color saturation, and edges. The first three cues were also prescribed by Beurs (Beurs, 1692; Beurs, in press) in his recipes for painting cut-open lemons and oranges, showing how research on material perception can be complemented by historical art writings and by the implicit knowledge of painters.

## Supplementary Material

Supplement 1

Supplement 2

Supplement 3

Supplement 4
