# Supplementary material for: If painters give you lemons, squeeze the knowledge out of them. A study on the visual perception of the translucent and juicy appearance of citrus fruits in paintings
Source: J Vis. 2020 Dec 22;20(13):12. doi: 10.1167/jov.20.13.12 (PMC7757633; doi:10.1167/jov.20.13.12)
Supplement: Supplement 1 [file jovi-20-13-12_s001.pdf]

Figure S1. Numbered list of all the stimuli used for Experiments 1, 2 and 3. Each image in the list is linked to the relative museum repository website.

**1**	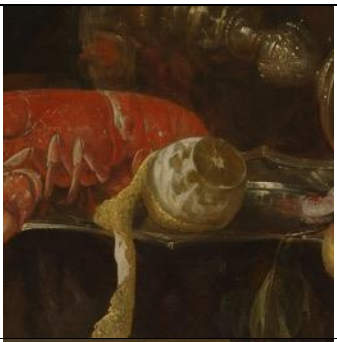	**2**	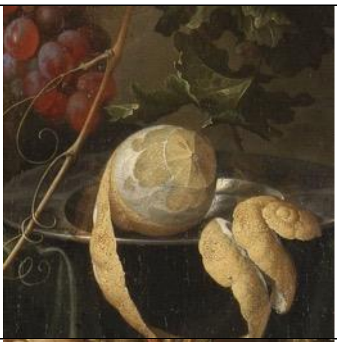	**3**	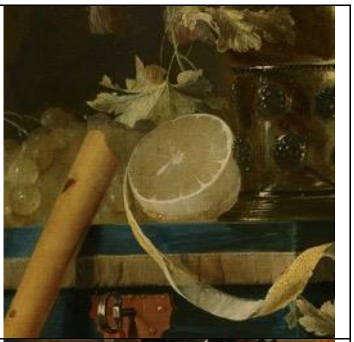
**4**	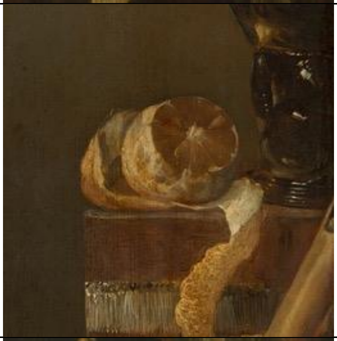	**5**	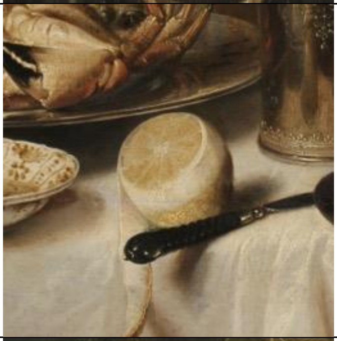	**6**	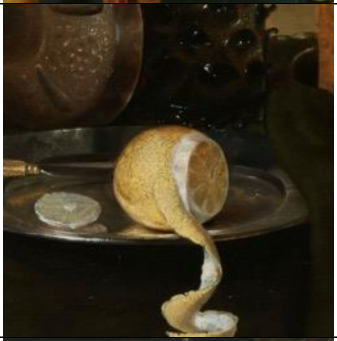
**7**	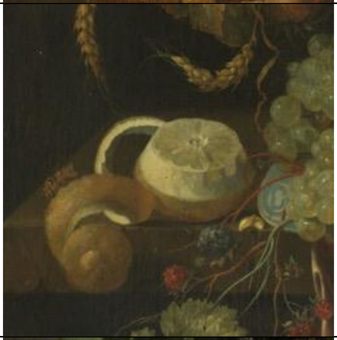	**8**	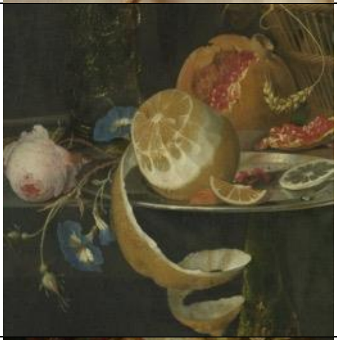	**9**	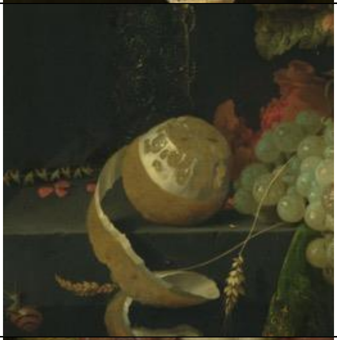
**10**	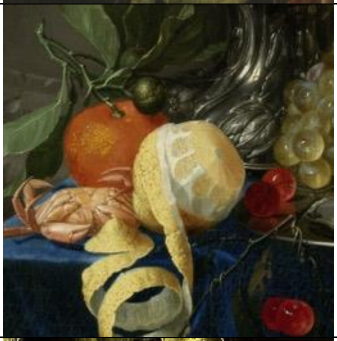	**11**	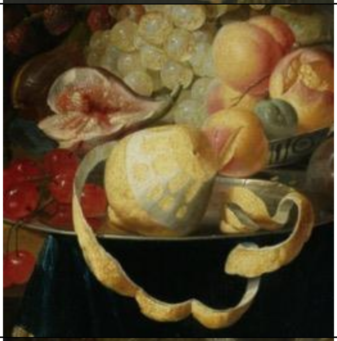	**12**	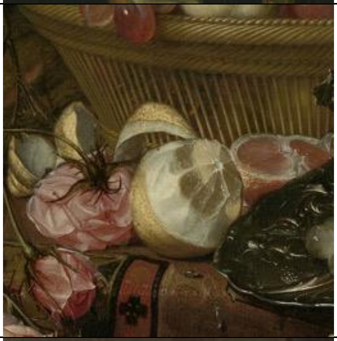
**13**	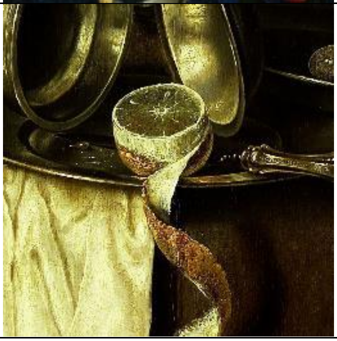	**14**	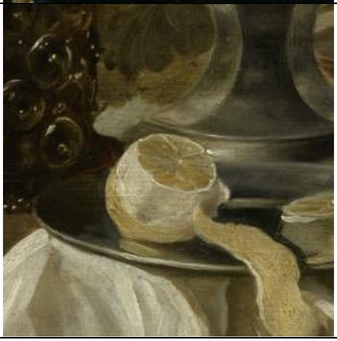	**15**	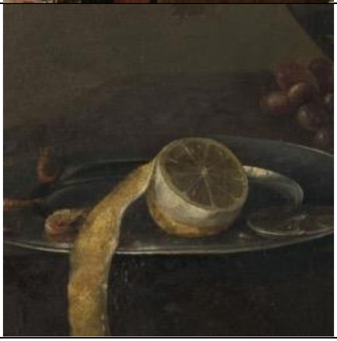

**16**	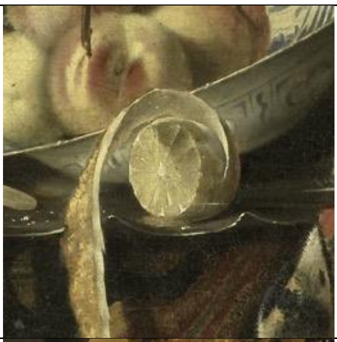	**17**	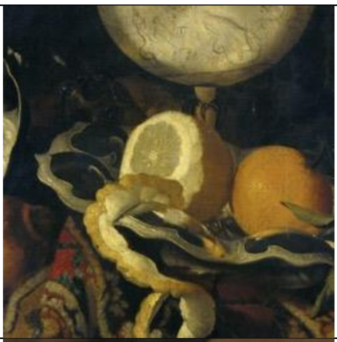	**18**	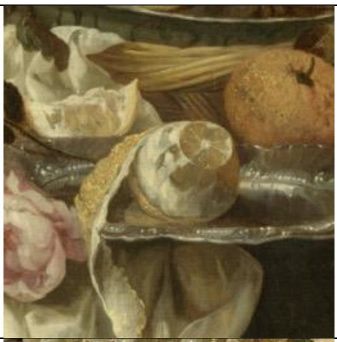
**19**	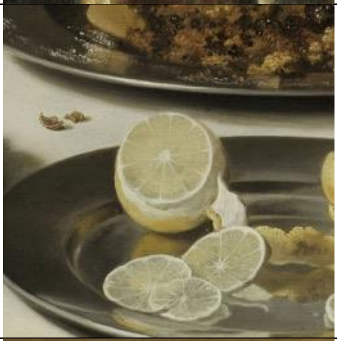	**20**	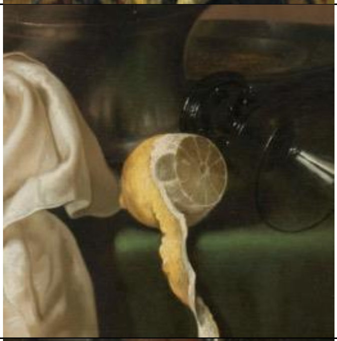	**21**	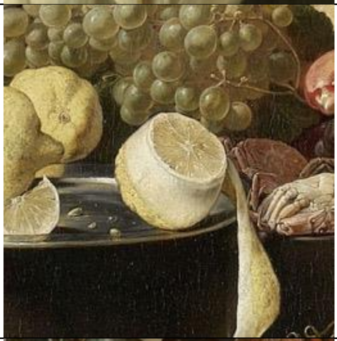
**22**	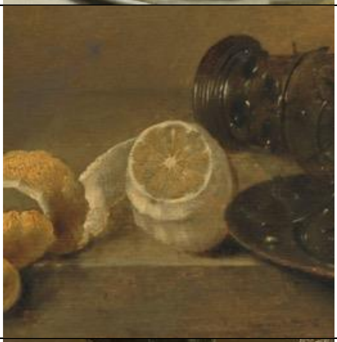	**23**	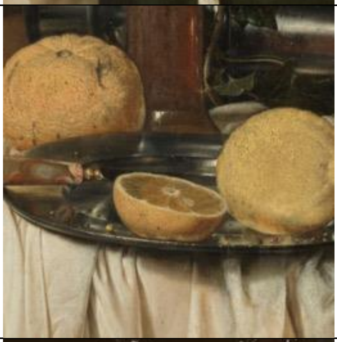	**24**	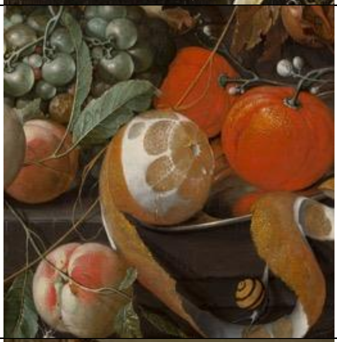
**25**	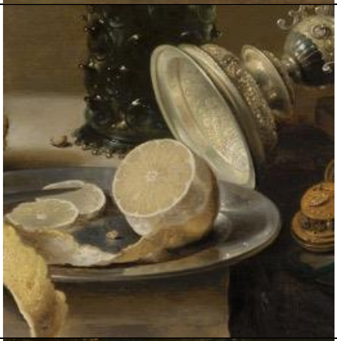	**26**	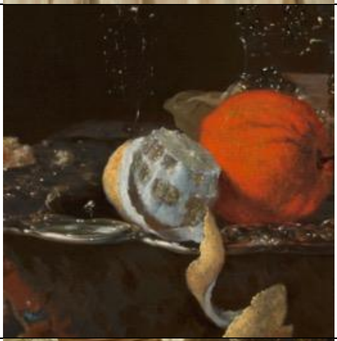	**27**	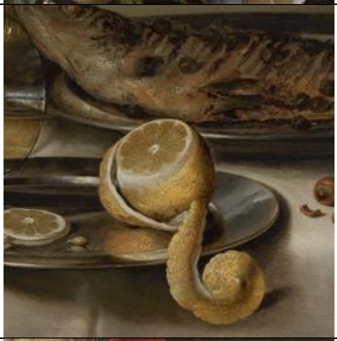
**28**	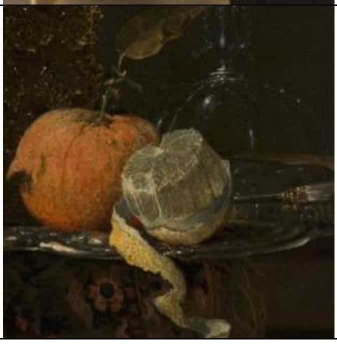	**29**	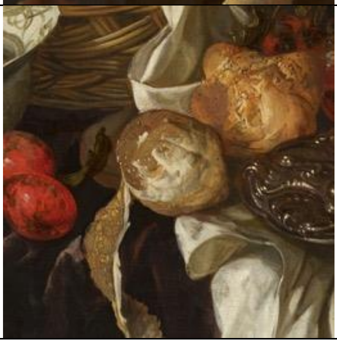	**30**	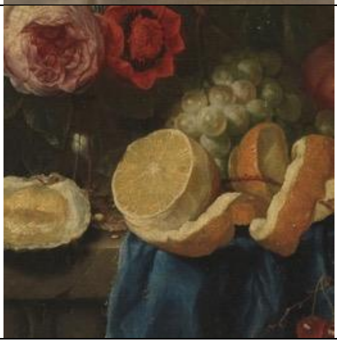

**31**	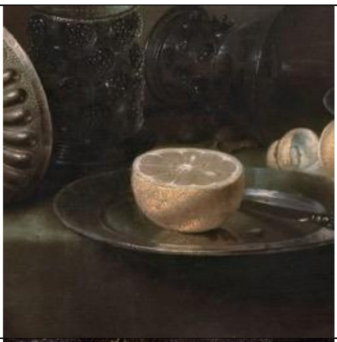	**32**	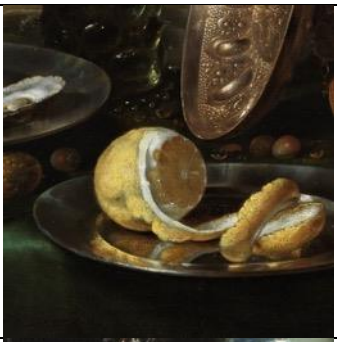	**33**	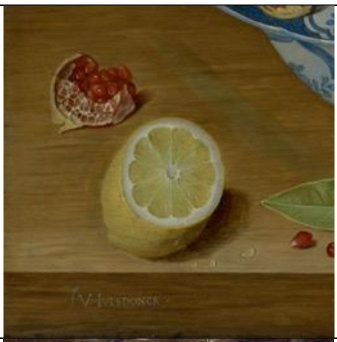
**34**	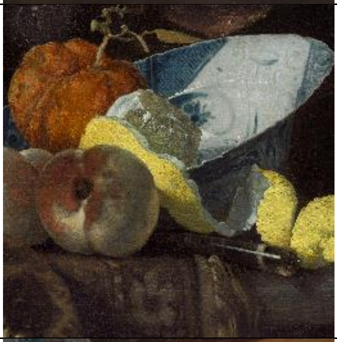	**35**	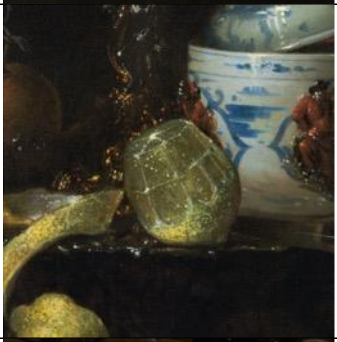	**36**	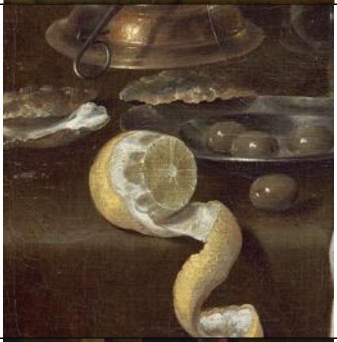
**37**	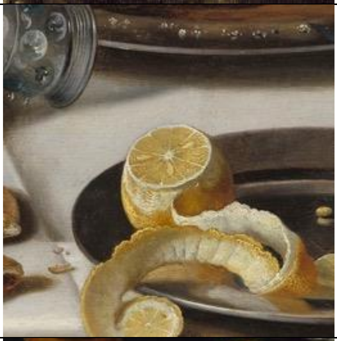	**38**	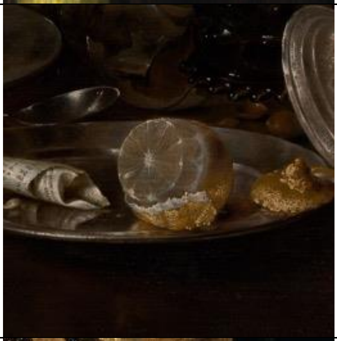	**39**	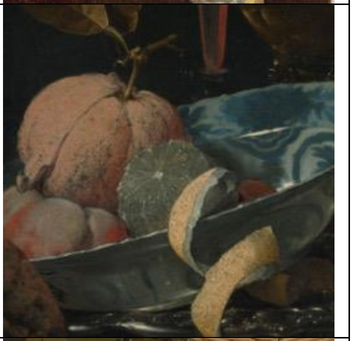
**40**	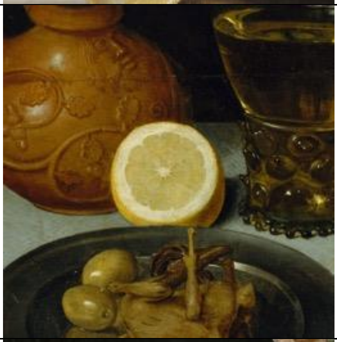	**41**	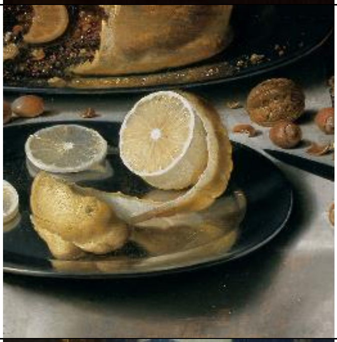	**42**	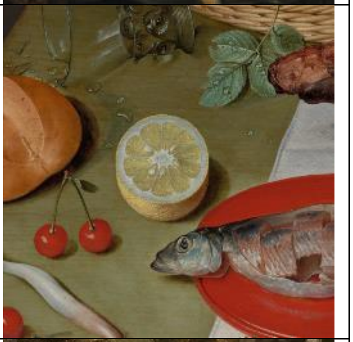
**43**	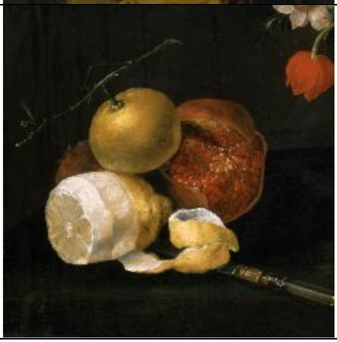	**44**	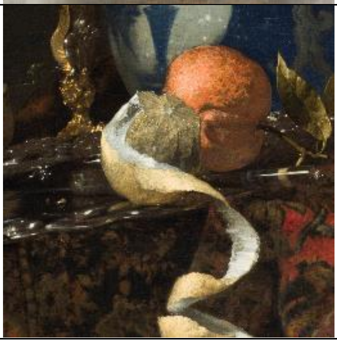	**45**	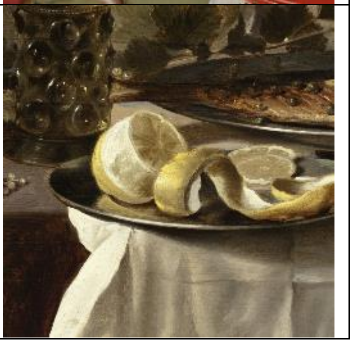

**46**	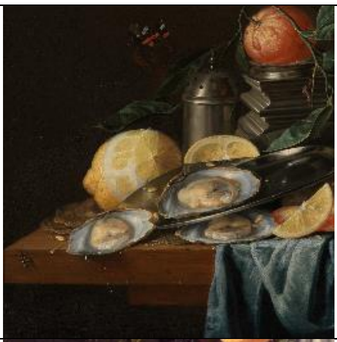	**47**	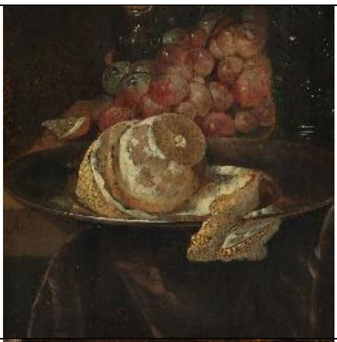	**48**	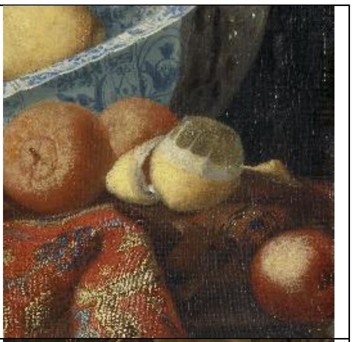
**49**	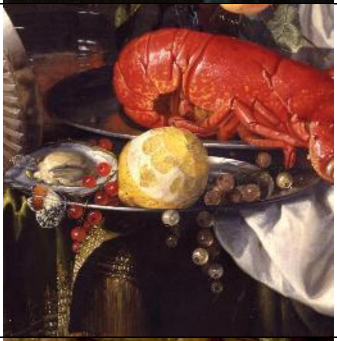	**50**	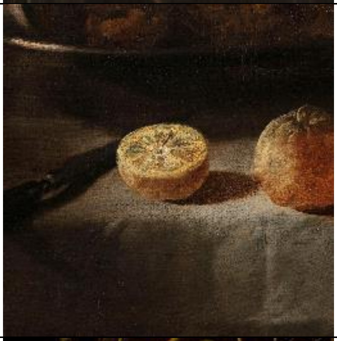	**51**	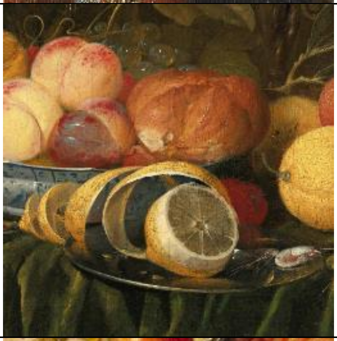
**52**	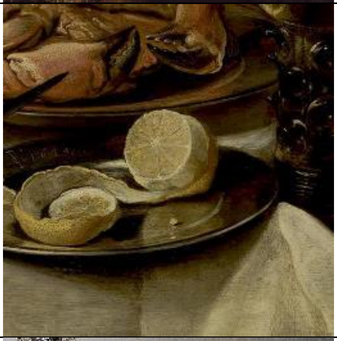	**53**	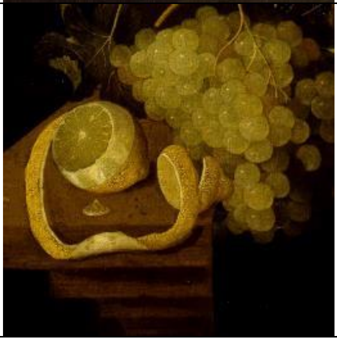	**54**	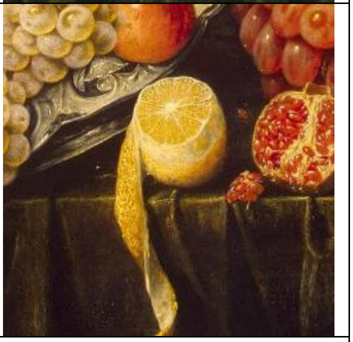
**55**	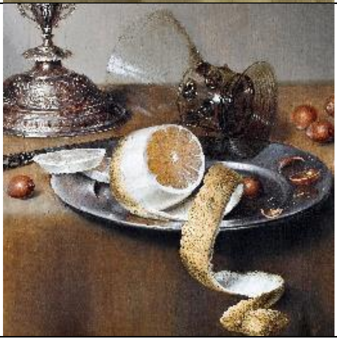				
