# Supplementary material for: If painters give you lemons, squeeze the knowledge out of them. A study on the visual perception of the translucent and juicy appearance of citrus fruits in paintings
Source: J Vis. 2020 Dec 22;20(13):12. doi: 10.1167/jov.20.13.12 (PMC7757633; doi:10.1167/jov.20.13.12)
Supplement: Supplement 2 [file jovi-20-13-12_s002.pdf]

S1. Instructions experiment 1

"How similar is the translucency/juiciness of the pulps of these citrus fruits?"

TRANSLUCENCY: Indicates that light can pass through the pulp of the citrus. It is the opposite of opaque.

JUICINESS: Indicates that the pulp of the citrus appears full of juice. It is the opposite of dry.
