# Supplementary material for: If painters give you lemons, squeeze the knowledge out of them. A study on the visual perception of the translucent and juicy appearance of citrus fruits in paintings
Source: J Vis. 2020 Dec 22;20(13):12. doi: 10.1167/jov.20.13.12 (PMC7757633; doi:10.1167/jov.20.13.12)
Supplement: Supplement 3 [file jovi-20-13-12_s003.pdf]

S2. Instructions experiment 2

"How big is the difference between the light and the dark part in the pulp of this citrus?"
LIGHT GRADIENT: magnitude of the change between the part of the pulp (surface + side when visible) in the light and the part of the pulp in the dark. Low values indicate that the light on the pulp looks uniform with no transition between light and dark; high values indicate that there is a big difference between the light and the dark parts of the pulp.

Uniform light

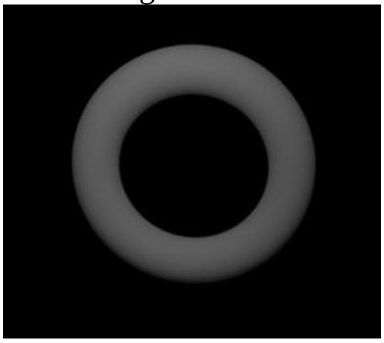

Light gradient

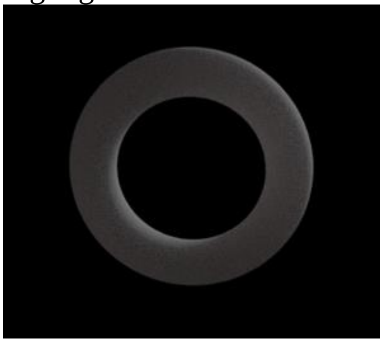

"*How sharp is the gradient between the light and dark part of the pulp surface of this citrus?"*
SHARPNESS OF GRADIENT: refers to the sharpness of the transition between the part of the pulp in the light and the part of the pulp in the dark. Low values indicate that the transition looks blurred; high values indicate that the transition looks very sharp.

Blurred gradient

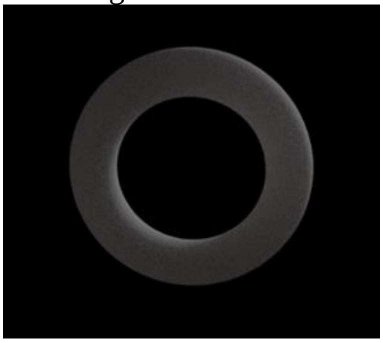

Sharp gradient

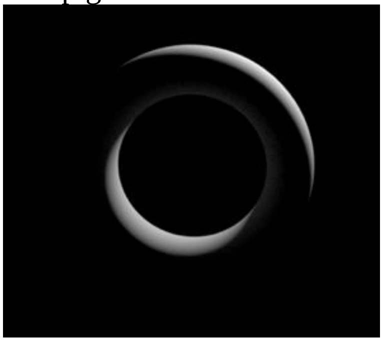

"How saturated is the color of the pulp of this citrus?"
COLOR SATURATION: defines the intensity of the color. Low values indicate that the color looks poorly saturated; high values indicate that the color looks highly saturated.

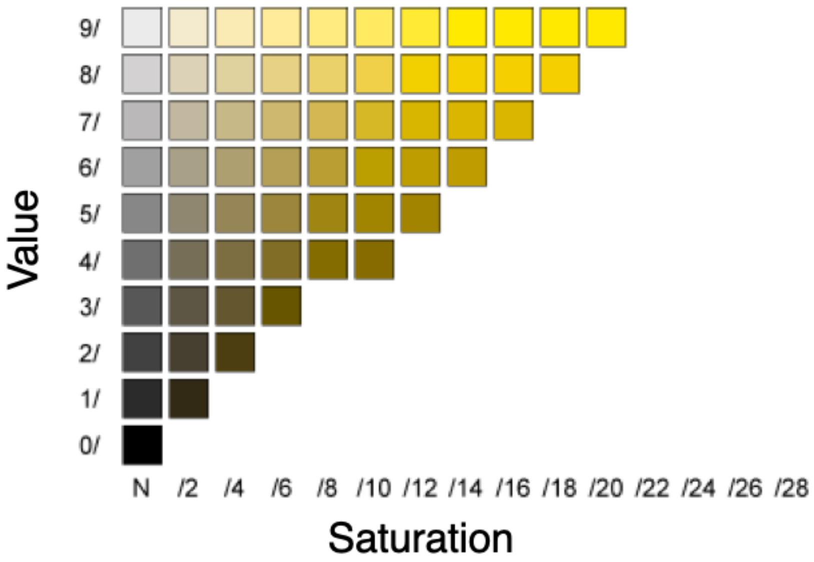

"How bumpy is the surface of the circular cross section of the pulp of this citrus?"
Low values indicate that the surface of the pulp looks completely flat; high values indicate that the surface of the pulp looks very bumpy. Bumpiness includes also the small cells in each slice.

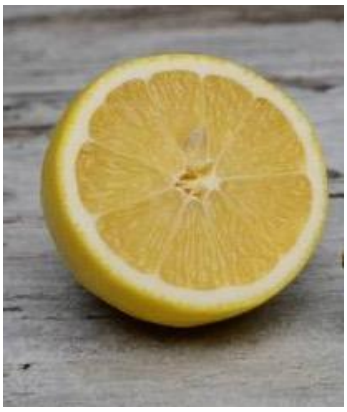

"Do you see highlights on the pulp of this citrus?"

"Do you see the seed(s) in the pulp of this citrus?"

"Do you see the (peeled) side of the pulp of this citrus?"
